# Atypical PKC: therapeutic target for Alzheimer’s?

**DOI:** 10.18632/aging.101730

**Published:** 2018-12-20

**Authors:** Robert V. Farese, Mini P. Sajan

**Affiliations:** 1 Division of Endocrinology and Metabolism, Department of Internal Medicine, University of South Florida, College of Medicine, Tampa, FL 33612, USA; 2 James A. Haley Veterans Administration Medical Center, Tampa, FL 33612, USA

**Keywords:** Alzheimer's disease, atypical PKC, beta-secretase, Abeta, phospho-tau

Dementias in the elderly are cruel and costly fates for afflicted patients and their families. Present therapeutic targets have had little success in preventing or treating these disorders.

In Alzheimer’s Disease (AD) and other dementias, Aβ plaques and phospho-tau tangles are the main pathological elements thought to impair neuronal functions of memory and cognition, and ultimately compromise neuronal integrity in the hippocampus and other critical brain areas. β-secretase activation is thought to be a key factor underlying the production of Aβ-peptide plaque precursors. Indeed, knockout of β-secretase can curtail AD development in transgenic mice harboring AD mutations [1]. Thus, much effort has been put into the development of effective and safe inhibitors of β-secretase, but, with limited success, there is a pressing need to develop alternate strategies.

Relevant to this need, we recently found that insulin acutely activated β-secretase and increased production of Aβ-peptides and phospho-tau in brains of intact mice, and in isolated neuronal cells and mouse hippocampal slices [2,3]. Moreover, hyperinsulinemia in several mouse and monkey models of insulin-resistant forms of obesity and type 2 diabetes mellitus (T2DM) apperared to be directly responsible for neuronal increases in β-secretase activity and levels of Aβ-peptides and phospho-tau; as correction of hyperinsulinemia by liver-directed treatments reversed these CNS aberrations [2,3]. These findings may help to explain how long-standing hyperinsulinemic states of obesity, the metabolic syndrome and T2DM, which collectively afflict over half of the over-fifty US population, may abet AD development.

Importantly, we found that the activation of atypical PKC (aPKC), PKC-λ/ι, rather than Akt [both protein kinases are activated by phosphatidylinositol 3-kinase (PI3K) during insulin action and mediate most insulin effects] was responsible for insulin-induced increases in β-secretase, Aβ-peptides and phospho-tau [2,3]. Perhaps more importantly, administration of aPKC inhibitors that pass the BBB were able to not only block acute insulin-dependent increases in β-secretase, Aβ-peptides and phospho-tau, but also, in mouse models of obesity and T2DM, simultaneously improve associated memory impairments [2,3]. As further evidence for aPKC importance, insulin-dependent increases in β-secretase, Aβ-peptides and phospho-tau are commensurately reduced in mice haploinsufficient for PKC-λ (unpublished).

However, insulin is but one of many factors known to activate aPKC. And, with respect to biological factors thought to abet AD development when present in excess or unduly activated, aPKCs are directly activated by tumor necrosis factor-α, ceramides (and therefore precursor fatty acids and spingolipids), phosphatidic acid (and therefore precursor fatty acids and glucose, and activators of phospholipase D, e.g., superoxide H_2_O_2_), and agents that, like insulin, activate tyrosine kinase receptors and PI3K, e.g., BDNF, NGF and AMPA receptor activators. This promiscuity of aPKC activation may provide a unifying mechanism whereby a wide variety of AD risk factors may abet AD development.

It may seem surprising to suggest that insulin excess serves as a risk factor for AD development, as it is commonly assumed that the brain is insulin-resistant in T2DM-associated AD, and the activity of the brain insulin receptor (IR) is modestly diminished in non-diabetic AD [4], Furthermore, nasal insulin, now in phase 3 clinical trials, is reportedly beneficial in some patients [5]. Note, however, that the existing hyperinsulinemia in high-fat-fed obese/T2DM mice markedly/fully increases brain IR activity, as well as activities of aPKC and Akt [3]. Also note that maximal activation of downstream processes are seen at submaximal levels of IR activation in most tissues (presumably in brain as well), i.e., there are “spare” IRs. Thus, as long as insulin levels are sufficiently elevated in insulin-resistant states, insulin may serve to increase aPKC activity, β-secretase activity, and levels of Aβ-peptides and phospho-tau. On the other hand, as insulin levels decline in T2DM, the brain may become hypo-insulinized and insulin therapy may have beneficial effects on apoptosis and various anabolic processes that are mediated by Akt.

Accordingly, there may be distinct phases of T2DM wherein the brain is hyper- or hypo-insulinized, and this may be relevant to successes and failures of insulin therapy in individual AD patients. Also, in normoinsulinemic non-diabetic AD, the observed decrease in brain IR activity [4] most likely limits insulin action, and insulin therapy may well be beneficial in these patients. In this regard, note that, although brain IR activity is mildly/modestly reduced in non-diabetic AD, the activities of aPKC and Akt are reportedly elevated [4], suggesting (a) the activation of PI3K (and thus aPKC and Akt) by non-insulin factors, and (b) that the reduction of IR activity in non-diabetic AD is due to well-recognized negative feedback inhibition of the IR via activation of aPKC, Akt, and/or other signaling factors, e.g., conventional PKCs, ERK and mTOR, that can function downstream of PI3K. Thus, in non-diabetic AD, whereas some elements of insulin action may be diminished by IR impairment, aberrations mediated by aPKC, may be excessive via action of non-insulin activator. This combination of impaired IR activity and elevated aPKC activity may benefit from a combination of insulin and aPKC inhibitor therapy.

To summarize, aPKC hyperactivity in brain appears to be present in both hyperinsulinemic phases of obesity and T2DM that may produce or abet AD development, and normoinsulinemic non-diabetic AD. Accordingly, the development of agents that diminish aPKC activity directly or indirectly may provide new approaches for preventing or treating various forms of AD and other dementias. This idea urgently needs to be tested.
